# Inflammatory Signaling and Brown Fat Activity

**DOI:** 10.3389/fendo.2020.00156

**Published:** 2020-03-24

**Authors:** Farah Omran, Mark Christian

**Affiliations:** ^1^Warwick Medical School, University of Warwick, Coventry, United Kingdom; ^2^Department of Biosciences, School of Science and Technology, Nottingham Trent University, Nottingham, United Kingdom

**Keywords:** inflammation, brown adipose tissue (BAT), cytokine, beige adipocyte, white adipose tissue

## Abstract

Obesity is characterized by a state of chronic inflammation in adipose tissue mediated by the secretion of a range of inflammatory cytokines. In comparison to WAT, relatively little is known about the inflammatory status of brown adipose tissue (BAT) in physiology and pathophysiology. Because BAT and brown/beige adipocytes are specialized in energy expenditure they have protective roles against obesity and associated metabolic diseases. BAT appears to be is less susceptible to developing inflammation than WAT. However, there is increasing evidence that inflammation directly alters the thermogenic activity of brown fat by impairing its capacity for energy expenditure and glucose uptake. The inflammatory microenvironment can be affected by cytokines secreted by immune cells as well as by the brown adipocytes themselves. Therefore, pro-inflammatory signals represent an important component of the thermogenic potential of brown and beige adipocytes and may contribute their dysfunction in obesity.

## Introduction

Obesity is generally associated with a systemic low-grade inflammation with adipocytes able to produce and release signaling proteins that contribute to this condition (1–4). Many pathologies are associated with this inflamed state, including cancer, heart disease, type 2 diabetes (T2DM), and neurodegenerative diseases. Additionally, inflammation has been shown to impact the function of BAT with thermogenic activity inhibited by TNFα-induced insulin resistance and proinflammatory cytokines secreted from macrophages (5–8).

Adipose tissue (AT) functions as the body's main organ to maintain energy homeostasis (9). Mammals have two main classes of AT; brown AT (BAT) and white (WAT) that act together to maintain a balance between fat accumulation and energy expenditure (10). AT is characterized by the presence of mature lipid-storing adipocytes and pre-adipocytes (11). However, it is heterogeneous in nature and composed of a wide range of additional cell types including macrophages, neutrophils, lymphocytes, endothelial cells, and nerve endings (12–14). With its secretion of over 100 different adipokines, cytokines, and chemokines, AT is the largest endocrine organ and links metabolism and immunity (15). Undesirable changes in adipokine expression including up-regulation of inflammatory markers and down-regulation of adiponectin are linked to obesity (16).

BAT dissipates energy through the process of non-shivering thermogenesis (10). This is facilitated by a large number of mitochondria, which express high levels of UCP1 (uncoupling protein 1) in the inner membrane (10). In brown adipocytes, the nucleus occupies a central position and triglycerides (TGs) are stored in many small multilocular lipid droplets (LDs) (17, 18). This provides a large LD surface accessible to lipases, which facilitates the rapid lipid consumption for adaptive thermogenesis (18). In contrast, WAT acts as a storage repository with white adipocytes maintaining TG in a single large LD that occupies a central position (16, 19). Its nucleus is located on the periphery and the cell possesses fewer mitochondria than brown adipocytes (16). Adipocytes with brown characteristics located within WAT are known as BRITE (brown-in-white) or beige adipocytes, and are found under conditions such as in response to cold or other stimuli (20–22). Evidence indicates that beige adipocytes are mostly derived from a different cellular lineage to that of classical brown adipocytes and have the capacity to reversibly transition between white and beige adipocytes (23). Due to the widespread prevalence of obesity and its associated diseases, there is considerable research interest in factors that modulate BAT thermogenesis and the beige phenotype to enhance weight loss and reduce morbidity risk. BAT itself has recently being recognized to practice an endocrine role. It can secrete multiple factors which could contribute to the systemic consequences of BAT activity. This also forms an interesting aspect of obesity research as it could lead to the identification of novel brown fat factors to direct drug discovery approaches and ultimately improve metabolic health (24).

The presence of BAT is associated with metabolic health and the amount of BAT is reduced in obesity (25–29). Higher BAT content and activation, such as by BAT transplantation in mice, positively affects glucose and insulin metabolism and body mass and plays a protective role against obesity pathogenesis and associated metabolic disorders such as hyperglycaemia and hyperlipidaemia (30–38). Cold induced thermogenesis, glucose uptake rates and insulin stimulation is severely impaired in BAT in the adiposity state (29, 39–41). Despite the studies showing that reduced BAT in obesity is associated with many negative metabolic consequences, understanding of the underlying mechanisms is limited. Chronic inflammation represents an important mechanism behind the dysfunction of BAT and browning of white adipocytes in obesity.

## Inflammatory Cells in Brown Adipose Tissue

Although mainly composed of brown adipocytes and pre-adipocytes BAT also contains a variety of immune cells such as macrophages, neutrophils and lymphocytes (42–44). Inflammation due to infiltration by macrophages and other immune cells is recognized as a key contributor to WAT pathophysiology in adiposity including insulin resistance and other alterations in metabolism (45, 46). Recent studies have identified infiltrated immune cells in BAT and inflammatory processes as contributors to BAT dysfunction in obesity and associated metabolic disorders. Similar to WAT, it is thought that recruitment of immune cells in BAT is a result of lipolysis and the release of fatty acids from stored TG (47). In diet-induced obese mice, after 6 months, BAT presents an increase in immune responses, including genes that indicate broad infiltration of leukocytes, monocytes, M1-macrophages, and cytokine release (48–51). However, BAT appears to be more resistant to macrophage infiltration than WAT in diet induced obese mice as these cells take longer to appear and have a more limited influence on BAT (50, 51). Also, the expression of inflammatory markers is lower in BAT than WAT regardless of diet (52) providing further support that BAT is generally more resistant to inflammation. Ultimately, inflammatory changes and higher expression of inflammation markers (including TNFα and F4/80) are evident in BAT after a persistent high burden of calorie intake (39, 52–54).

Enhanced inflammation is suggested to play a major role in the whitening of BAT that occurs after prolonged exposure to high fat diet at thermoneutrality. This transformation of brown adipocytes to unilocular cells similar to white adipocytes, is a result of a combination of various factors that include triggering macrophage infiltration, brown adipocyte death, and crown-like structure (CLS) formation. Whitened BAT shows CLS formation surrounding adipocytes that contain enlarged endoplasmic reticulum, cholesterol crystals, some degenerating mitochondria, and become surrounded by an increased number of collagen fibrils. BAT gene expression analysis shows that whitened BAT is associated with a strong inflammatory response and activation of nucleotide-binding oligomerization domain-like receptor-3 inflammasome (NLRP3) (72). In addition, the multimodular adaptor protein p62 is involved in multiple functions including inflammation, and it contributes to regulating energy metabolism via control of mitochondrial function in BAT which is another indicator of the importance of inflammation and immune cells pathways in BAT biology (73).

The enhancement in BAT inflammation is considered to be largely a result of the existence and active participation of infiltrated pro-inflammatory immune cells which are listed and reviewed below:

## Macrophages

Macrophages are immune cells that serve an important role in the coordination of inflammatory processes (74). Classically activated macrophages (M1) secrete high levels of pro-inflammatory cytokines including TNF-α, MCP-1, IL-1β, and IL-6, whereas alternatively activated macrophages (M2) produce anti-inflammatory cytokines including IL-4 (2). In subcutaneous fat (scWAT), positive roles are reported for M2 macrophages in adaptive thermogenesis. Adipocyte-derived adiponectin signals to activate M2 macrophage proliferation during chronic cold exposure and the depletion of macrophages or adiponectin leads to resistance to cold-induced browning in scWAT (75). M2 macrophage activation also contributes to the beiging effects of adrenomedullin 2 (ADM2) and subsequent increased UCP1 expression in adipocytes (76). ADM2 can be produced by white adipocytes, and its expression is down-regulated in adipose tissues of obese mice (76). M2 macrophage activation is also stimulated by meteorin-like (Metrnl) supporting the link between adaptive thermogenic responses and anti-inflammatory gene programs in fat (77).

In the lean state BAT resident macrophages which are mostly the M2 subtype (78, 79). In obesity, however, BAT is infiltrated with (M1) macrophages which are suggested to play a crucial role in controlling adaptive thermogenesis. Inflammation of BAT caused by infiltrated macrophages reduces thermogenesis and UCP1 activation (39, 43). However, how macrophages affect thermogenesis and BAT biology is controversial (78). Initially, cold-induced thermogenesis was thought to be dependent on the secretion of the cytokines IL-4 and IL-13 by innate lymphoid cells and eosinophils that signal to macrophages as deletion of these cytokines receptors was found to diminish UCP1 expression and heat generation (77, 80). It was also suggested that M2 macrophages participate in this mechanism by secreting catecholamines (77, 80–82). However, this concept was recently challenged (83). It was found that adipose resident macrophages do not express tyrosine hydroxylase (the rate limiting enzyme for the catecholamine synthesis) and chronic treatment of wild type, UCP1^−/−^, and IL-4 receptor knockout mice with IL-4 failed to increase energy expenditure. In addition, incubation of adipocytes with conditioned medium from IL-4 stimulated macrophages did not induce UCP1 protein expression (83). These data indicate that any role of macrophages in brown fat activation is not through IL-4 stimulated secretion of catecholamines. However, a role of macrophages should not be completely ruled out in thermogenesis.

The main pathway for thermogenesis activation in BAT is via the sympathetic nervous system. It has recently been demonstrated that macrophages play a role in the control of BAT innervation; as selective depletion of the nuclear transcription factor Mecp2 (methyl-CpG- binding protein 2) in macrophages, a murine model of Rett syndrome, leads to spontaneous obesity with compromised homeostatic energy expenditure and thermogenesis of BAT. Specifically, deficiency of Mecp2 in BAT-macrophages causes a reduction of UCP1 gene expression levels that appears to result from impaired sympathetic innervation (43). Moreover, adipose tissue resident macrophages are reported to express a set of genes, or have a subpopulation attached to sympathetic neurons, which regulate norepinephrine levels by controlling its degradation which influences adipose tissue thermogenesis (84, 85).

## Mast Cells

Mast cells are immunological classic mediators of allergic reactions and the main secretors of histamine (86). They are present in both WAT and BAT and their number increases in obesity (87, 88). Similar to some macrophages, they are closely associated with the vasculature (88). Brown adipocytes have high levels of histamine contained in mast cells and it is reported to play a role in thermogenesis through the H2-receptor. This action appears to be independent of any effect on noradrenaline stimulated oxygen consumption in isolated brown adipocytes (89). In response to colder temperatures, mast cells secrete histamine, IL-4 and other factors that promote UCP1 expression and the beiging response of WAT (90). Furthermore, it is proposed that acute cold exposure recruits mast cells to the WAT of lean subjects and enhances their degranulation and histamine secretion in both lean and obese subjects. As degranulation positively correlates with UCP1 suggests thermogenesis and beiging enhancement through histamine and secretion of other factors (91). However, these positive associations between mast cells and thermogenesis/beiging of WAT has been challenged. Zhang et al. reported that mast cell deficiency or pharmacological inhibition in mice increases browning of WAT by increasing beige adipocyte differentiation. It has also been demonstrated that mast cell-derived serotonin inhibits WAT browning and systemic energy expenditure (92). The mouse model used for this study has a mutation in c-kit tyrosine kinase and a degree of caution in the interpretation of the outcomes is required. Several alternative (c-kit-independent) genetic models of mast cell depletion have found that there is essentially no effect of mast cells in obesity and related pathologies. That is because diet-induced obese mice with either deficiency or proficiency of mast cells exhibits similar profiles of weight gain, glucose tolerance, insulin sensitivity, metabolic parameters, and AT or liver inflammation (93, 94). Further research is needed to fully understand the role of mast cells in brown and beige adipocytes especially in humans.

## T Lymphocytes: T_reg_ and ILC2s Cells

T_reg_ cells are a small subset of T lymphocytes and are considered to be one of the most crucial defense mechanisms in maintaining appropriate immune responses including roles in autoimmunity and inflammation (95). T_reg_ cells appear to be reduced in obesity and also required to maintain a normal adaptive thermogenesis response to cold (96, 97). Depletion of this type of immune cell impairs BAT function which was demonstrated by decreased oxygen consumption and prevention of the activation of thermogenic genes coincident with enhanced inflammation and the invasion of proinflammatory macrophages (96).

ILC2s (IL-33/Group 2 innate lymphoid cells) are a subtype of innate lymphoid cells. ILC2s are activated by epithelial cell-derived cytokines IL-33 and IL-25 as well as thymid stromal lympoiphoidin (TSLP) in response to allergens. In WAT, it has been found that white adipocytes themselves (98) and endothelial cells (99) can express IL-33. ILC2s control eosinophil and pro inflammatory macrophages to initiate type 2 immune responses that prevent helminth infection or promote pathologic allergic inflammation (100). They are found in WAT and their number is decreased in obese mice and humans (101). These cells essentially release IL-5 which maintains macrophage responses and IL-13 which controls eosinophil responses. Both of these cytokines appear to play an indirect role as mediators of beiging of WAT (100). In addition, ILC2s produce an opioid-like peptide, methionine-enkephalin (MetEnk) peptide, which appears to directly upregulate UCP1 in WAT and induces the beiging process (101). The cytokine IL-33 limits the development of spontaneous obesity by increasing numbers of ILC2s and eosinophils. This coincides with beiging and energy expenditure in the WAT of mice by but not BAT. Deletion of IL-33 leads to opposite effects (100, 102, 103).

## Impact of Inflammation and Inflammatory Mediators on Brown Adipocyte Function

Obesity mediated upregulation of inflammatory cytokines has been extensively studied in WAT, while relatively little is known about the cytokines involved in the adiposity inflammatory state in BAT and how it affects BAT function and thermogenesis. However, there is an increasing amount of evidence that inflammation directly alters the thermogenic activity of brown fat by impairing its energy expenditure mechanism and glucose uptake. Pro-inflammatory cytokines can affect thermogenesis in BAT (104–106) and also determine the capacity of WAT browning (106, 107). It has been clearly demonstrated that infiltrated macrophages and other immune cells in subcutaneous WAT negatively impact the ability of precursor cells to differentiate into thermogenically active beige adipocytes because of pro-inflammatory cytokine secretion and generating an inflammatory microenvironment (108).

In BAT, the increased expression of inflammatory markers such as TNFα and MCP-1 in obese murine models is accompanied by a decrease in the expression of UCP1 and other markers of thermogenesis as well as lack of fatty acids which are needed as substrate for thermogenesis (49, 109). Also, it is reported that IL-1b reduces the cAMP-mediated induction of UCP1 expression (104), cold-induced thermogenesis in adipocytes *in vivo* via sirtuin-1 inhibition (SIRT1) (110) and WAT browning (111). Furthermore, Fractalkine, which is an adipocyte-synthesized chemokine, appears to contribute to enhancement of the pro-inflammatory status of BAT and reduced thermogenic gene expression in diet-induced obese mice (59). In contrast, IL-13, which has anti-inflammatory properties, induces GDF15 (growth differentiation factor 15) expression which is found to protect against obesity by inducing thermogenesis, lipolysis, and oxidative metabolism in mice (112, 113), and prevent inflammation through inhibition of M1 macrophage activation (71).

Oncostatin M, a macrophage proinflammatory cytokine impairs BAT thermogenesis and browning capacity of subcutaneous WAT *in vivo*. Furthermore, it inhibits brown adipocyte differentiation *in vitro* (114, 115). The pro-inflammatory phenotype induced by Oncostatin M is indicated as a mechanism of downregulating UCP1 expression. The significance of inflammation-driven inhibition of beige adipogenesis in obesity has been highlighted by studies of the interaction between α4-integrin receptor on pro-inflammatory macrophages and VCAM-1 (vascular cell adhesion molecule-1) on adipocytes. This interaction resulted in reduced UCP1 gene expression via the ERK (extracellular signal-regulated kinase) pathway and blockage of α4 integrin led to elevated beige adipogenesis and prevented metabolic dysregulation of the obese AT (113). This mechanism establishes a self-sustained cycle of inflammation driven impairment of the beige phenotype in obesity.

Another inflammatory candidate that might affect BAT biology and thermogenesis is the macrophage secreted factor GDF3 (growth differentiation factor-3) which increases in obesity. It is suggested that GDF3 is responsible for inhibition of β3-adrenoceptors which can lead to reduced lipolysis and consequently the impaired release of fuel for thermogenesis (84). However, although thermogenic gene expression was not restored after deleting activin-like kinase-7 (Alk7) which is the GDF3 receptor (84, 116, 117), deleting Alk7 led to reduced obesity. The metabolic benefit of Alk7 deletion may be attributed to enhanced mitochondrial biogenesis and increased levels of fatty acid oxidation found in this mouse model (116) as browning did not occur.

BAT can respond to immune and inflammatory pathways by the expression of cytokine receptors, Toll like receptors (TLRs), and nucleotide-oligomerization domain-containing proteins (NODs). Activation of these receptors by immune and metabolic signals mediates a negative impact of proinflammatory signaling on BAT thermogenesis (53). In this respect, both LPS and TNFα are found to impair UCP1 in BAT in mice *in vivo* and *in vitro* studies (110, 118). In addition, TLR4 activation inhibits β3-adrenergic-induced browning of WAT, whereas TLR4-deletion maintains thermogenic capacity (111). Some inflammatory inducers can lead to greater disruption of WAT browning compared to affecting thermogenesis in BAT. This may be indicative of a greater inflammatory response of WAT compared BAT. For example, depletion of the intestinal microbiota leads to greatly enhanced WAT browning while having only a minor effect on typical BAT (119). Also, LBP (LPS-binding protein) depletion similarly enhances WAT browning (120). This might be explained by a higher basal level of inflammation in subcutaneous WAT compared to BAT (51, 106, 107).

On the molecular level, IKKε (IκB kinase ε) and IRF3 (interferon regulatory factor-3) are among the main inflammation regulators in obesity (121, 122). Deletion of IKKε or IRF3 results in a reduction of inflammatory markers in adipose tissues and enhanced WAT browning with UCP1 expression and energy expenditure increased, while there are only minor effects on BAT (122, 123). The Nod-like receptor 3 (NLRP3) inflammasome multiprotein complex regulates inflammation and macrophage activity by cleaving IL-1b and IL-18 precursors into their active forms. Activation of NLRP3 in macrophages attenuates UCP1 and adaptive thermogenesis induction of white adipocytes and mitochondrial respiration, while NLRP3 deletion prevents UCP1 reduction. The action is through IL-1 as blocking the IL-1 receptor in adipocytes protected thermogenesis activity (111). IEX-1, an immediate early gene, is highly expressed in macrophages in obesity and is responsible for the majority of the obesity associated inflammation in humans and mice and its deletion had profound effects on the browning of WAT. Knockout of IEX-1 prevents HFD-induced inflammation, insulin resistance, and obesity by elevated browning and increasing thermogenic gene expression in WAT. This results from the promotion of M2 macrophages in WAT, but not BAT (124): and further highlights the different immune responses of white and brown adipose tissues.

There is a link between inflammatory stress pathways and the accompanied activation of endoplasmic reticulum (ER) in conditions of disruption of systemic metabolic homeostasis like obesity (125, 126). Brown adipocytes have a relatively small ER content and restricted ER surface area compared to other cell types. Thus, ER adaptation in these cells may require alternative pathways to conventional mechanisms such as chaperone-mediated protein folding and ER expansion (127, 128). In fact, some of the canonical ubiquitin-proteasome system molecules, for example X-box binding protein 1, appear to be dispensable in adipocytes (129). To maintain ER homeostasis and cellular integrity increased proteasomal activity in brown adipocytes is reported to be essential for thermogenic adaptation. This occurs via induction of the induction of the ER-localized transcription factor nuclear factor erythroid-2, like-1 (Nfe2l1, also known as Nrf1) (130). Deletion of Nfe2l1, specifically in brown adipocytes, results in ER stress, inflammation, mitochondrial dysfunction, insulin resistance, and whitening of the BAT (130, 131).

In addition to the direct effects that proinflammatory cytokines may have on brown adipocytes, some of these factors may inhibit activation of adrenergic receptors, stimulation of sympathetic nervous activity and thus local secretion of noradrenaline. As this is the main mechanism of inducing BAT thermogenesis activity and WAT browning, in response to cold and diet, it should be considered when evaluating effects on browning.

## Brown Adipocytes Secrete Pro/Anti-Inflammatory Mediators

In addition to the cytokine mediators secreted by infiltrated immune cells, such cytokines may also be secreted by brown adipocytes themselves. Table 1 summarizes what has been studied in BAT.

**Table 1:** Summary of brown adipocyte secreted inflammatory mediators.

**Inflammatory mediator**	**Role in brown/beige adipocytes**	**Inflammation**		**Cold-regulated**	**References**
		**Pro-**	**Anti-**		
Chemerin	↑ Chemerin expression in brown adipocytes in obesity ↑ Chemerin gene expression in brown adipocytes through differentiation Chemerin predicted to increase triglyceride accumulation	✓		↓	(55)
Endothelin 1 (ET-1)	ET-1 inhibits adipogenesis Adrenergic activation inhibits ET-1 secretion	✓ (?)		↓	(56)
Retinol-Binding Protein 4 (RBP4)	↑ RBP4 expression in BAT with thermogenic, noradrenergic activation	✓ (?)		↑	(57)
Growth differentiation factor (GDF8/myostatin)	Myostatin leads to ↓ thermogenesis and browning and ↓ metabolic activity in BAT	✓		(?)	(58)
Classic pro-inflammatory cytokines such as MCP1, TNFα, IL-1.	The increase in these cytokines is accompanied with ↓ thermogenesis genes and ↓ mitochondrial respiration in BAT	✓		↓	(53)
Fractalkine (CX3CL1)	Enhanced CX3CL1 secretion leads to ↑ pro-inflammatory status and ↓ thermogenesis gene expression in BAT	✓		(?)	(59)
Insulin-Like Growth Factor-1 (IGF-1)	IGF-1 leads to ↑ proliferation and differentiation of preadipocytes	✓	✓	↑	(60, 61)
IL-6	↑ IL-6 expression in BAT with adrenergic stimulus	✓	✓	↑	(62)
Fibroblast growth factor 21 (FGF21)	FGF21 leads to ↑ thermogenesis		✓	↑	(63, 64)
Follistatin (Fst)	Fst leads to ↑ thermogenesis and browning Fst leads to ↑ adipocyte differentiation		✓	↑	(65)
C-terminal fragment of SLIT2 protein (SLIT2-C)	SLIT2-C leads to ↑ browning		✓	↑ (acute)	(66)
				- (Chronic)
C-X-C motif chemokine ligand-14 (CXCL14)	CXCL14 leads to ↑ browning and ↑ (M2) macrophages in BAT		✓	↑	(67)
Vascular endothelial growth factor A (VEGFA)	VEGFA leads to ↑ thermogenesis and browning		✓	↑	(68, 69)
Lipocalin prostaglandin D synthase (L-PGDS)	L-PGDS leads to ↑ basal metabolic rates and ↑ lipid utilization in BAT		✓	↑	(70)
Growth and differentiation factor 15 (GDF15)	↑ GDF15 gene expression and release with noradrenergic, cAMP-mediated thermogenic activation of brown adipocytes Inhibits local inflammatory pathways originated from macrophages		✓	↑	(71)

**Chemerin** is an adipokine associated with inflammation markers (e.g., IL-6, TNFα, Leptin) and components of the metabolic syndrome in WAT. It modulates chemotaxis and activation of dendritic cells and macrophages (132–134). Chemerin was found to be secreted by brown adipocytes. Its gene expression levels are increased in obesity and decreased with cold induced thermogenesis and could potentially play a key role as an inflammatory modulator in BAT. However, the lack of correlation between expression levels in BAT and circulating levels make it unclear whether it plays an endocrine role in attracting immune cells (55). At present, it remains to be determined how Chemerin expression is controlled and what is its function in BAT.

**Endothelin-1 (ET-1)** has pro-inflammatory effects by activating macrophages, resulting in the secretion of pro-inflammatory and chemotactic mediators including TNFα, IL-1, IL-6, and IL-8 (135, 136). ET-1 levels were found to be increased in obesity and enhance lipolysis thereby linking it to insulin resistance in WAT (137). ET-1 is released by brown adipocytes and its secretion is inhibited during adrenergic stimulation (56). Data implicates that it can inhibit thermogenesis via induction of Gq signaling. However, the contribution of ET-1 to inflammation of BAT and the mechanism of thermogenesis repression remains to be fully investigated.

**Vascular endothelial growth factor A** (VEGFA) is a proangiogenic cytokine. The reported findings concerning VEGFA levels in WAT in obesity are controversial. Deletion of VEGFA in WAT leads to little or no change in the expression of inflammatory markers that contribute to systemic insulin resistance, such as EGF-like module-containing mucin-like hormone receptor-like 1 (Emr1), TNF-α, and MCP-1. Nor were there detectable changes in the expression of mitochondrial genes in WAT (138, 139). In contrast, in BAT VEGFA can induce thermogenic activity and deletion of VEGFA results in reduction of BAT mass, vessel density, and eventually loss of thermogenesis through mitochondrial dysfunction (68, 138). Hence, ablation of VEGFA results in the whitening of BAT. However, the direct effect of VEGFA on inflammatory markers in brown adipocytes is currently unknown.

**Retinol-Binding Protein 4** (RBP4) is an adipokine and circulating transporter of vitamin A (retinol) that induces inflammation and promotes the secretion of proinflammatory molecules (140). There has been some controversy regarding the associations and/or causality in the context of obesity and metabolic syndrome. However, in adipocytes, RBP4 appears to have a relevant role in obesity and the development of insulin resistance and diabetes (141). Brown adipocytes release RBP4 when exposed to a thermogenic, noradrenergic activation, but the mechanism associated with this release is unclear. Lipocalin 2 (Lcn2) has been implicated in the release of RBP4 as Lnc2 KO adipose tissue shows increased RBP4 levels while circulating levels are reduced (142). Also, BAT released RBP4 may not be associated with insulin resistance given that cold-induced activation of BAT is associated with insulin sensitization (57).

**Fibroblast growth factor 21 (FGF21)** is a brown adipokine and a key factor in the regulation of energy homeostasis. In WAT, it induces browning and participates in improving glucose metabolism and weight regulation. Cold induced thermogenesis and adrenergic activation induces FGF21 release from brown adipocytes. In addition to this mechanism, WAT-resident anti-inflammatory invariant natural killer T (iNKT) cells promote the release of FGF-21 by adipocytes and the browning process (143–145). Prevention of hyperglycaemia and hyperlipidaemia is associated with high levels of FGF21 in line with high BAT activity and enhanced energy expenditure (146, 147). Although FGF21 is reported to have anti-inflammatory effects on white adipocytes (148) it remains to be determined if it has a similar action in brown adipocytes.

**CXCL14** is a member of the CXC chemokine family and exerts chemoattractive activity for activated macrophages, immature dendritic cells and natural killer cells. In WAT, CXCL14 participates in glucose metabolism (149, 150). It is reported to be secreted by brown adipocytes in response to thermogenic activation. CXCL14 appears to attract M2 macrophages and its deletion leads to impaired BAT thermogenesis activity and low recruitment of macrophages into BAT. CXCL14 enhances the browning of white fat via type 2 cytokine signaling (67).

**Fractalkine (CX3CL1)** is a chemokine produced by brown adipocytes that plays a role in the recruitment of leukocytes through the fractalkine receptor. Its action seems to be to promote an inflammatory state as deficiency of the fractalkine receptor prevents BAT accumulation of macrophages and leads to reduced expression of pro inflammatory genes (Tnfα, Il1α, and Ccl2) in mice exposed to HFD. Furthermore, the BAT of fractalkine receptor deficient mice shows increased expression of lipolytic enzymes such as adipose triglyceride lipase (Atgl), lipase, hormone-sensitive (Hsl) and monoglyceride lipase (Mgtl) and upregulation of UCP1 and other thermogenesis genes (59). This indicates that fractalkine serves a key role in the local inflammation of BAT tissue and the remodeling on HFD affecting metabolism.

**Follistatin (FST)** is a soluble glycoprotein that has the capacity to modulate the activities of multiple members of the transforming growth factor (TGF) family, specifically activin A and myostatin (GDF8). TGF-β superfamily cytokines play pivotal roles in regulation of tissue functions including inflammation (151–153). Blockade of TGF-β/Smad3 signaling enhances insulin sensitivity and prevents diet-induced obesity, promotes the browning of WAT with reduced levels of inflammatory cytokines and less inflammatory macrophage infiltration (154–156). FST is upregulated in BAT in response to cold, and is potentially a positive regulator of BAT function by blocking TGF-β signaling pathways, GDF8 actions, and exerting anti-inflammatory effects (65, 157, 158). However, these actions are yet to be explored.

**Myostatin (GDF8)** is a key member of the transforming growth factor-β (TGF-β) super family and has an essential role in the regulation of overall fat content in mice. Loss of GDF8 leads to a significant increase in lean mass, total energy expenditure, protection against diet-induced obesity, and insulin resistance. GDF8 levels increase in obesity and it is reported to suppresses Irisin leading to activation of inflammatory cytokines and insulin resistance in WAT. GDF8 secretion from brown adipocytes is stimulated by activation of hunger-related neural circuits. It negatively regulates BAT thermogenesis as well as WAT browning, and metabolic activity. Data clearly indicates that GDF8 inhibits brown fat gene expression (157), however, further research is needed to investigate its inflammatory related effects in this tissue (58, 156, 157, 159–161).

**Growth and differentiation factor 15 (GDF15)** is also known as macrophage inhibiting cytokine-1 and is a member of the TGF-β superfamily (162). GDF15 is suggested to be a reliable predictor of disease progression in certain tumors, inflammatory diseases, cardiovascular disease, and obesity (162). It is reported to decrease food intake, body weight and adiposity, and to improve glucose tolerance under normal and obesogenic diets (163). Furthermore, systemic overexpression of GDF15 was shown to prevent obesity and insulin resistance by increasing the expression of the main thermogenic and lipolytic genes and oxidative metabolism in BAT and WAT (164). It is identified as one of the factors secreted by brown adipocytes through protein kinase A-mediated mechanisms, and highly induced in response to thermogenic activity following stimulus by cold, norepinephrine, and cAMP. GDF15 acts on macrophages in BAT and may mediate inhibition of local inflammatory pathways under conditions of enhanced BAT activity (71).

**C-terminal fragment of SLIT2 protein (SLIT2-C)** belongs to the Slit family of secreted proteins that play important roles in various physiologic and pathologic activities including inflammatory cell chemotaxis where it exercises an anti-inflammatory role (66, 165). SLIT2-C expression is regulated by PRDM16 and is secreted from beige/brown adipocytes. It induces thermogenesis, WAT browning, and metabolic processes associated with substrate supply to fuel thermogenesis. The pathway for the induction of thermogenesis is independent of β-adrenergic activation, but requires activation of protein kinase A signaling (66). The protease that generates the SLIT2-C fragment as well as the receptor in BAT that binds it are important areas for future investigation.

**Lipocalin prostaglandin D synthase (L-PGDS)** is expressed in BAT where it has a key role in energy substrate utilization. It is also localized in the central nervous system and it is involved in inflammatory modulations amongst other functions (166). Deletion of L-PGDS leads to inadequate thermogenesis in BAT because of impairment in switching of substrate utilization from glucose to lipids (70, 167). In addition, L-PGDS deficiency induces obesity possibly through the regulation of inflammatory responses (168). However, if that is the case in brown adipocytes, it is yet to be elucidated.

**Insulin-Like Growth Factor-1 (IGF-1)** appears to play pleiotropic functions and provides signals to macrophages to sustain adipose tissue development and homeostasis. IGF1 signaling integrates immune-metabolic interactions to facilitate macrophage activation status. Cold exposure stimulates elevation of IGF-1 expression in the BAT of rats (169). However, Myeloid-specific ablation of IGF-1 receptor worsens diet induced obesity but not cold induced thermogenesis (170, 171). It is suggested that IGF-1 is released by brown adipocytes and involved in proliferation and differentiation of brown preadipocytes (60, 61, 172). IGF-1 upregulation due to BAT transplantation is proposed to abolish type I diabetes in this experimental model and negatively correlates with glucose, glucagon, and inflammatory cytokines in rodents (31, 173). However, the detailed role of IGF-1 in brown adipocytes inflammation regulation remains an area to be investigated.

**Figure 1:**
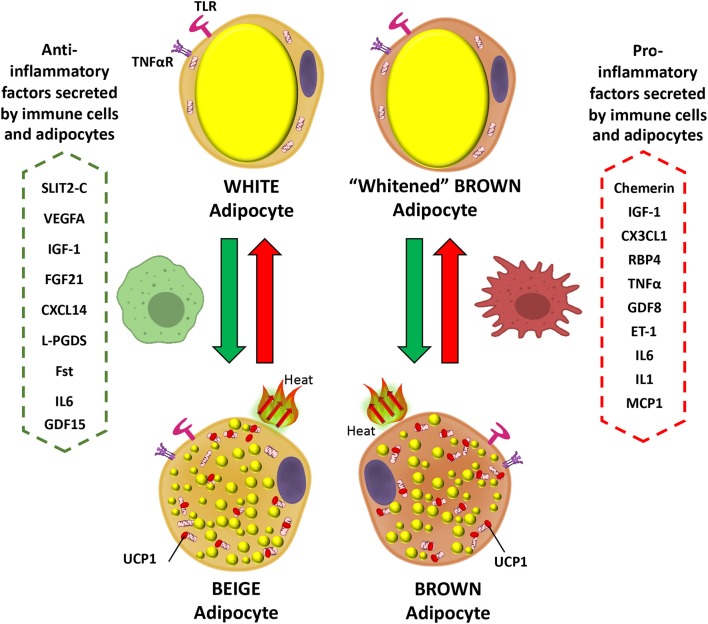
Inflammatory mediator actions on white and brown adipocytes. Pro-inflammatory factors secreted by immune cells and brown/beige adipocytes prevent the expression of brown fat genes in adipocytes, including UCP1, the main thermogenic protein (red arrows). In contrast anti-inflammatory mediators promote the transition of white to beige adipocytes and could prevent expression of the “whitened” brown adipocyte phenotype in brown adipose tissue (green arrows). IGF-1, Insulin-Like Growth Factor-1; CX3CL1, Fractaline; RBP4, Retinol-Binding Protein 4; TNFα, Tumor necrosis factor a; GDF8, Growth differentiation factor 8; ET-1, Endothelin 1; IL6, Interleukin 6; IL1, Interleukin 1; MCP1, Monocyte Chemoattractant Protein-1; SLIT2-C, C-terminal fragment of SLIT2 protein; VEGFA, Vascular endothelial growth factor A; FGF21, Fibroblast growth factor 21; CXCL14, C-X-C motif chemokine ligand-14; L-PGDS, Lipocalin prostaglandin D synthase; Fst, Follistatin; UCP1, Uncoupling Protein 1; GDF15, Growth and differentiation factor 15.

**Interleukin 6 (IL-6)** is secreted by brown adipocytes upon β-adrenergic activation (62). Chronic elevation of IL-6 in the CNS leads to increased UCP1 in BAT, but not in denervated BAT tissue which suggest a central role in IL-6-dependent promotion of thermogenesis (174). Signaling by IL-6 promotes M2 macrophage polarization in BAT (175). Evidence indicates that IL-6 is released from WAT during the differentiation of human beige adipocytes to facilitate the commitment of adipocyte precursors toward beigeing and enhancement of thermogenesis capacity in an autocrine manner (176). The deletion of IL-6 in mice leads to inefficient BAT transplantation with sustained obesity and insulin resistance, and blunted FGF21 increase (33). These data suggest beneficial effects of IL-6 in regulation of BAT metabolism possibly directly or indirectly related to FGF21 actions. However, this contrasts with the action of IL-6 as a potent pro-inflammatory cytokine. This aspect can be demonstrated by plasma IL-6 being elevated in obesity and diabetes, in addition to reduced levels in weight loss (177–179). Furthermore, it is also found to play a major role as a pro-inflammatory cytokine in obese adipose tissue, macrophage polarization, and T cell regulation via STAT3, leading eventually to insulin resistance and worsening diet-induced obesity (180). Moreover, as expected for IL-6 having a typical role as a pro-inflammatory cytokine, its deletion causes reversal of pro-inflammatory signaling in the obese state (179). In terms of browning activation, IL-6 is implicated in inducing inguinal WAT atrophy by accelerating WAT lipolysis and browning (181). In any case, these available contradictory data concerning the role of IL-6 are indicative of both pro- and anti-inflammatory actions. A schematic showing the actions of inflammatory mediators on brown and white adipocytes is presented in Figure 1.

## Insulin Sensitivity

Pro-inflammatory signaling can disrupt the insulin signaling cascade and impair the insulin sensitivity of BAT. Although, IL-1, TNF-α, MIF, and IL-6 have consistently been shown to cause insulin resistance in WAT (182, 183), their effects have not been extensively explored in BAT at the molecular level. Elevated inflammatory marker levels in the diet induced obesity state in mice are suggested to be responsible for BAT insulin resistance via AKT (protein kinase B) and ERK pathways (52). TNF-α appears to play an important role in impairing insulin sensitivity of BAT. The mechanism has been discussed in detail and it involves disturbances of both MAP- kinases activation and IRS-2 and AKT (5–7). Mammalian target of rapamycin complex 2 (mTORC2), which activates inflammation, sustains thermogenesis via Akt-induced glucose uptake and glycolysis in BAT. This highlights the significance of glucose metabolism in BAT in thermogenesis and indicates the importance of identifying how inflammation can affect mTORC2-activation in BAT (184, 185). Alleviating the inflammation state in obesity may restore insulin sensitivity as targeting inflammation in diet induced obesity in mice leads to a decrease in adipocyte area, macrophage infiltration, proinflammatory gene expression, along with JNK and NF-κB activation and increased insulin sensitivity via increased AKT phosphorylation (186, 187). In this context, sucrose non-fermenting related kinase (SNRK), a member of the AMPK-related kinase family, is found to suppress inflammation in WAT and is essential for maintaining UCP1 expression for BAT thermogenesis. Dysregulation of this anti-inflammatory kinase leads to induction of insulin resistance in BAT via impairment of the PP2A-Akt pathway (188, 189). As a result, inflammation is a modulator of insulin responses in BAT and is strongly linked to UCP1 expression and thermogenesis. It is important to determine the role of each inflammatory cytokine in insulin resistance and thermogenesis in order to identify therapeutic targets.

## Mitochondrial Function Is Affected by Inflammatory Pathways

Inflammation and mitochondrial dysfunction are closely linked with obesity and associated with alteration in mitochondrial function and mass (190, 191). These alterations are further demonstrated by downregulation of mitochondrial biogenesis, oxidative metabolic pathways, and oxidative phosphorylation proteins in WAT in obesity, and a negative correlation with pro-inflammatory cytokines (192). Evidence indicates that proinflammatory cytokines have a significant influence on modulating mitochondrial efficiency leading to effects on energy homeostasis in human white adipocytes. TNF-α most dramatically alters 3T3-L1 adipocyte mitochondrial functions, whereas IL-1β and IL-6 have more modest effects. Moreover, activation of the NLRP3 inflammasome in macrophages attenuates UCP1 induction and mitochondrial respiration in cultures of primary adipocytes possibly via IL-1, while the absence of NLRP3 is protective for UCP1 and adaptive thermogenesis capacity in adipocytes (111, 193, 194).

The activation of pattern recognition receptors in brown adipocytes and subsequent increased inflammation leads to mitochondrial dysfunction and suppression of mitochondrial respiration with reduced UCP1 expression levels and repressed white adipocyte browning capacity in response to adrenergic stimulation. Mechanistically, these effects are likely to involve inhibition of SIRT1 activity (53, 110, 111). Moreover, deletion of TLR4 protected mitochondrial function and thermogenesis in WAT (111). However, there is a suggestion that mitochondrial dysfunction in adipocytes is a primary cause of adipose tissue inflammation, adipocyte enlargement and insulin resistance. According to this hypothesis mitochondrial dysfunction and fatty acid oxidation in adipocytes leads to adipocyte enlargement because of triglyceride accumulation. Furthermore, adipocyte mitochondrial dysfunction leads to pseudo-hypoxia with greater accumulation of hypoxia-inducible factor 1α (HIF-1α), which elevates adipose tissue inflammation and fibrosis (195, 196). Similarly, alteration of mitochondrial capacity in BAT might be functionally associated with defective thermogenesis and energy expenditure in obesity and increased risk to develop obesity-induced insulin resistance.

Using a mouse model of chronic systemic inflammation, which exhibits increased circulating levels of inflammatory cytokines and abnormal regulation of both innate and adaptive immune responses, mitochondrial swelling is detected with severe damage of the cristae, in addition to reduced cold-induced thermogenic capacity and UCP1-dependent mitochondrial respiration (197). Furthermore, low grade inflammation in BAT in obesity is found as a contributor to excess reactive oxygen species (ROS) production and associated oxidative stress, which may cause mitochondrial dysfunction (198–202). Further investigations in BAT confirmed increased inflammation and ROS generation, but this was accompanied by the doubling of mitochondria respiration compared to lean subjects. It is possible that if the obesogenic conditions were maintained for longer, mitochondria would have eventually failed to deal with obesity stress and thermogenic capacity would be ultimately compromised (49). ROS production does not necessarily have negative consequences in BAT. Consistent with beneficial effects of increased ROS, activated BAT thermogenesis *in vivo* is defined by a substantial increase in mitochondrial ROS levels and pharmacological depletion of mitochondrial ROS leads to hypothermia upon cold exposure, and inhibits UCP1-dependent increases in whole body energy expenditure (203).

Mitochondrial dysfunction resulting from deletion of the mitochondrial transcription factor A (TFAM) leads to adipocyte death coincident with inflammation in WAT and a whitening of BAT with decreased energy expenditure. BAT whitening in these mice is mainly explained by impairment of mitochondrial electron transport chain function, reduced fatty acid oxidation, and increased circulating fatty acids, rather than a conversion of brown to white adipocytes (204). These findings highlight the link between mitochondrial function and inflammation and point to mitochondria dysfunction leading to increased inflammation which could ultimately lead to a vicious cycle.

## Anti-Inflammatory Pathways and Bat Function

Strategies that target the inflammatory status may have the potential to reverse adipose tissue dysfunction and prevent progression of metabolic diseases. Suppression of inflammation using pharmacological agents, with reduction of pro-inflammatory cytokines and macrophage infiltration in WAT, improves AKT-phosphorylation in response to insulin along with improved body weight and fat mass (187, 205–207). Cytarabine, which has immunosuppressive actions, is associated with enhanced BAT activity via the AMPK pathway raising the possibility it could be developed for anti-obesity therapy (208). There is also evidence that dietary intervention can have anti-inflammation activity which leads to enhanced insulin sensitivity. Food extracts with a high content of either flavonoids, phenolic compounds, p-coumaric acid, quercetin, or resveratrol have been found to exert systemic anti-inflammatory actions via inhibition of TNF-α-triggered activation of MAPKs and NFκB in human white adipocytes which improve insulin sensitivity (186). Specifically, Curcumin intervention was found to reduce mouse WAT inflammation and increase BAT UCP1 expression via PPAR-dependent and -independent mechanisms. It reduces macrophage infiltration and proinflammation cytokine expression in both macrophages and adipocytes along with increased energy expenditure and body temperature in response to cold (209).

Fatty acids (FA) are another example of dietary constituents that act as inflammation modulators. Importantly, ω3-FAs (n-3 polyunsaturated fatty acids (n-3PUFAs) have anti-inflammatory effects and may significantly impact chronic inflammatory diseases including obesity related disorders (210). An ω3-enriched diet, in non-obesogenic non-inflammatory conditions, leads to synthesis of oxylipins which have an anti-inflammatory response in both WAT and BAT with a macrophage modulation effect, but with no influence on inflammatory cytokine secretion (209). FFAs are active stimulators for members of the rhodopsin-like family of G protein-coupled receptors (GPCRs) including GPR40, GPR41, GPR43, GPR84, and GPR120 (211, 212). GPR120 is highly expressed in both BAT and WAT. and positively impacts metabolic health by stimulating mitochondrial respiration in brown fat via intracellular Ca^2+^ release which results in mitochondrial depolarization and fragmentation. This occurs along with mitochondrial UCP1 activation, which may act synergistically with mitochondrial fragmentation to increase respiration. GPR120 activation by the agonist TUG-891 upregulates fat combustion in BAT thereby reducing fat mass, while GPR120 deficiency diminishes expression of genes involved in nutrient metabolism (213). Moreover, GPR120 deficiency leads to obesity, glucose intolerance, and hepatic steatosis in mice fed a high-fat diet (214). Importantly, GPR120 mediates the anti-inflammatory and insulin sensitizing effects of ω3-FAs including inhibition of inflammatory pathways and cytokine secretion in adipocytes and macrophages (215, 216). A role for GPR120 in BAT activation and WAT browning in response to cold via FGF21 secretion has also been confirmed (217).

## Conclusion

Immune responses pose a significant metabolic challenge for the host due a range of energetically expensive processes including inflammatory mediator production and cell migration and proliferation. There is a trade-off between the energetic demands of immunity and homeothermy that permits a hypometabolic-hypothermic state to favor the immune system. Peripheral insulin resistance provides a mechanism for reallocating metabolic fuels to immune cells due to decreased nutrient storage in fat, muscle, and liver. The precise role of BAT in the hypometabolic-hypothermic state is currently unclear. Although BAT is generally more resistant to inflammatory stimuli than WAT, the repression of thermogenesis by inflammation may be a key energy trade-off to allow sufficient resources for immune responses. Importantly, BAT-mediated thermogenesis reactivation seems to be required for the exit from the hypometabolic-hypothermic state (218).

Many immune and inflammatory cells actively participate in the regulation of BAT thermogenesis, WAT browning and ultimately have the capacity to participate in controlling energy balance, glycemia, and lipidemia. Pro-inflammatory mediators secreted by both immune cells and adipocytes inhibit thermogenesis activation in BAT and browning of WAT in contrast to anti-inflammatory factors that have a positive influence. Additional research is needed to demonstrate the effect of each one of these mediators on brown and beige adipose cells and fully explain the pathways involved at the molecular level that regulate immune cells and brown/beige adipocytes interactions. This could lead to new therapeutic strategies to improve metabolic health and combating obesity and associated metabolic diseases.

## Author Contributions

All authors listed have made a substantial, direct and intellectual contribution to the work, and approved it for publication.

### Conflict of Interest

The authors declare that the research was conducted in the absence of any commercial or financial relationships that could be construed as a potential conflict of interest.
